# Supplementary figures and images for: Emergency department utilization for substance use disorders and mental health conditions during COVID-19
Source: PLoS One. 2022 Jan 13;17(1):e0262136. doi: 10.1371/journal.pone.0262136 (PMC8757912; doi:10.1371/journal.pone.0262136)

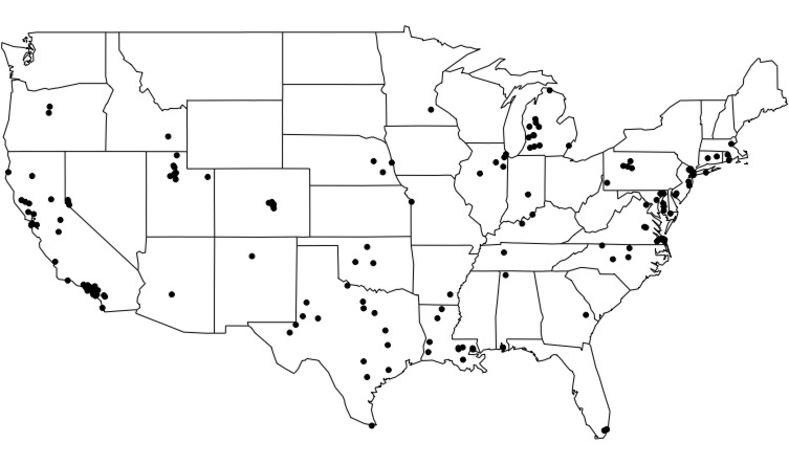

Supplement: S1 Fig — (TIF) [file pone.0262136.s001.tif]

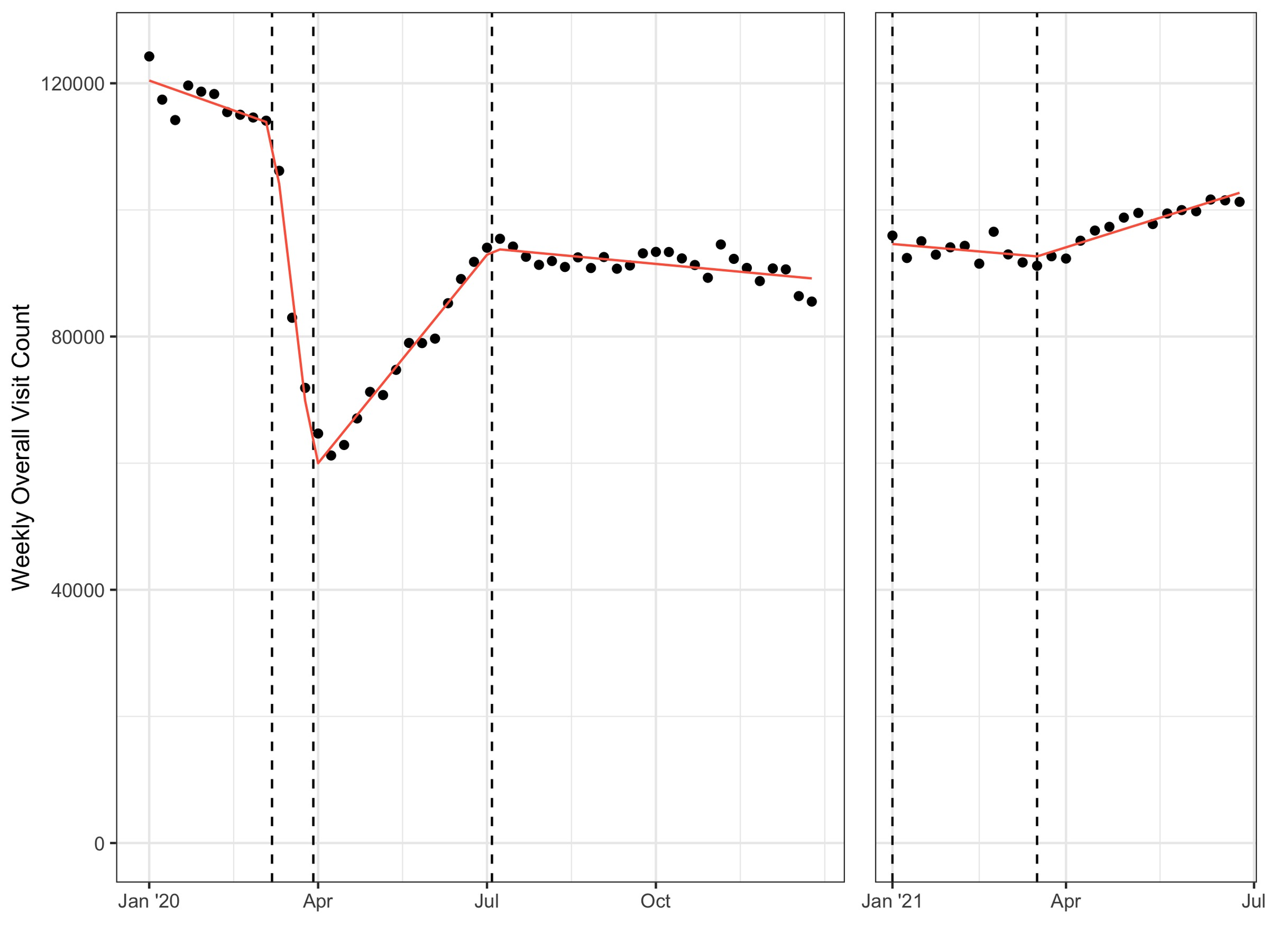

Supplement: S2 Fig — (TIF) [file pone.0262136.s002.tif]

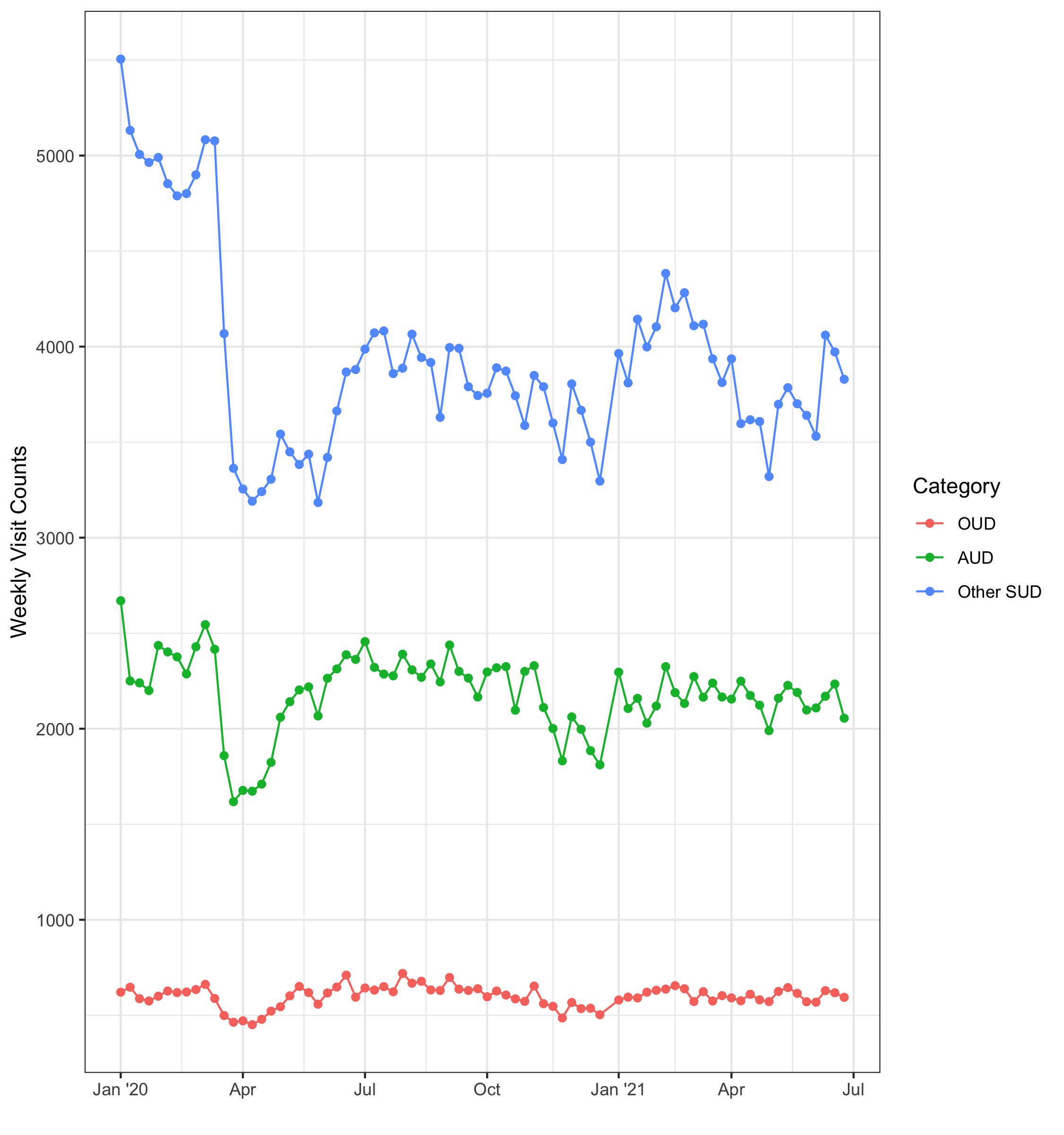

Supplement: S3 Fig — (TIF) [file pone.0262136.s003.tif]

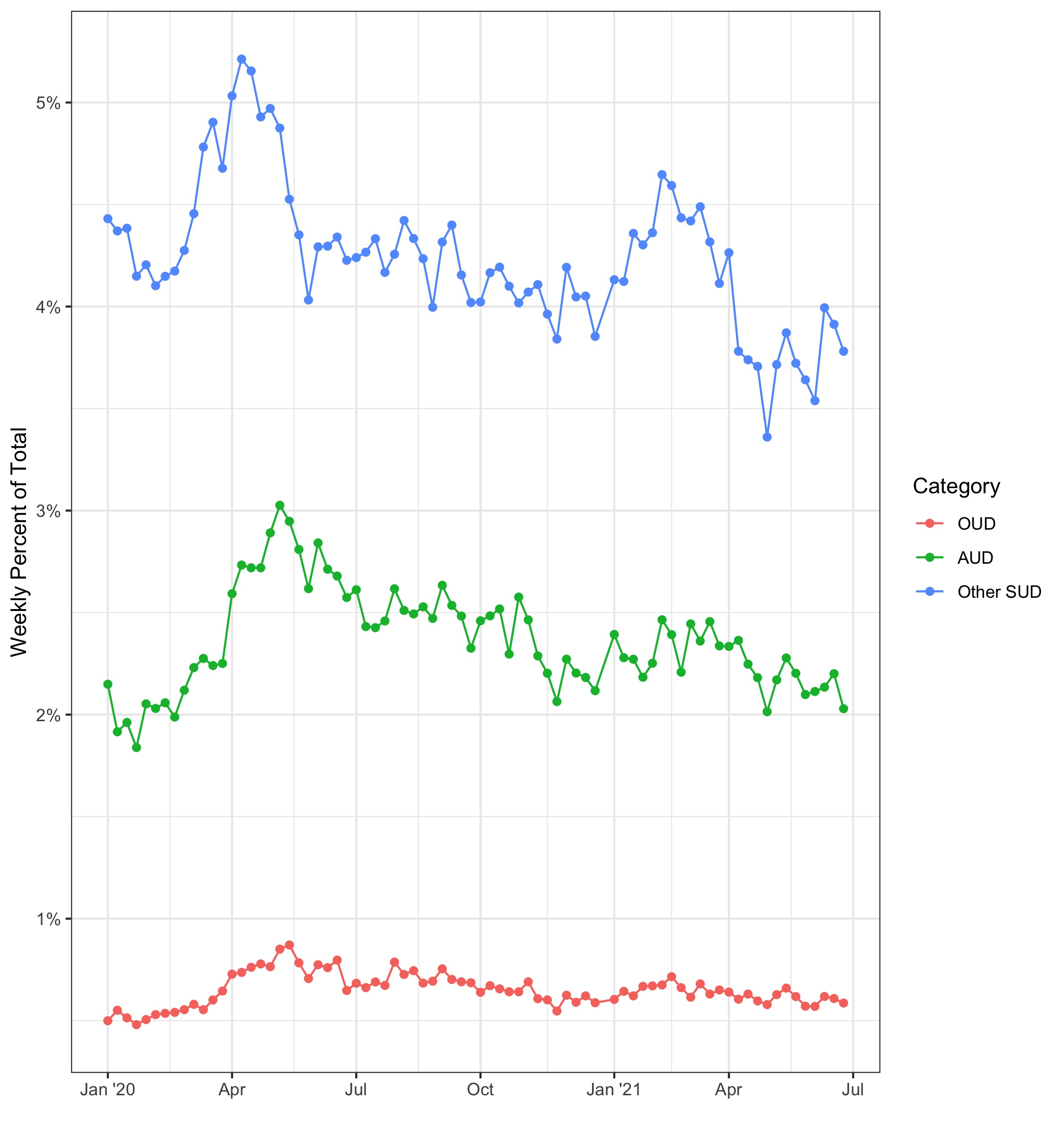

Supplement: S4 Fig — (TIF) [file pone.0262136.s004.tif]
