# Supplementary material for: Emergency department utilization for substance use disorders and mental health conditions during COVID-19
Source: PLoS One. 2022 Jan 13;17(1):e0262136. doi: 10.1371/journal.pone.0262136 (PMC8757912; doi:10.1371/journal.pone.0262136)
Supplement: S1 Table — (DOCX) [file pone.0262136.s005.docx]

| Substance-Use Disorder | CCSR | MBD017 | Alcohol-related disorders |
| --- | --- | --- | --- |
|  |  | MBD018 | Opioid-related disorders |
|  |  | MBD019 | Cannabis disorders |
|  |  | MBD020 | Sedative disorders |
|  |  | MBD021 | Stimulant disorders |
|  |  | MBD022 | Hallucinogen disorders |
|  |  | MBD023 | Inhalant disorders |
|  |  | MBD025 | Other specified substance-related disorders |
| Mental Health-Related | Any ‘Root’ ICD-10 Code | F2 | Schizophrenia, schizotypal, delusional, other |
|  |  | F3 | Mood (affective) disorders |
|  |  | F4 | Anxiety, dissociative, stress-related, somatoform, other nonpsychotic mental disorders |
|  |  | F5 | Behavioral syndromes associated with physiological disturbances and physical factors |
|  |  | F6 | Disorders of adult personality and behavior |
|  |  | F7 | Intellectual disabilities |
|  |  | F8 | Pervasive and specific developmental disorders |
|  |  | F9 | Behavioral, emotional disorders, unspecified disorders |
| Opioid Use Disorder | CCSR | MBD018 | Opioid-related disorders |
| Alcohol Use Disorder | CCSR | MBD017 | Alcohol-related disorders |
| Other Substance-Use Disorder | CCSR | MBD019 | Cannabis disorders |
|  |  | MBD020 | Sedative disorders |
|  |  | MBD021 | Stimulant disorders |
|  |  | MBD022 | Hallucinogen disorders |
|  |  | MBD023 | Inhalant disorders |
|  |  | MBD025 | Other specified substance-related disorders |
| MI and  Stroke | CCSR | CIR009 | Acute myocardial infarction |
|  |  | CIR020 | Cerebral infarction |
