# Supplementary material for: Emergency department utilization for substance use disorders and mental health conditions during COVID-19
Source: PLoS One. 2022 Jan 13;17(1):e0262136. doi: 10.1371/journal.pone.0262136 (PMC8757912; doi:10.1371/journal.pone.0262136)
Supplement: Attachment — Submitted filename: CEDR-OUD-PLOS-ONE-RESPONSE-REVIEWERS(1).docx [file pone.0262136.s006.docx]

**Rebuttal letter/Responses to PLOS Medicine Reviewers (responses in bulleted italics)**

Reviewer Notes:

Reviewer #1:

This paper uses electronic medical record data from a registry of 170 U.S. EDs (~3-4% sample of the ~5,000 Eds in the U.S.) spanning Jan 2019 - mid Nov 2020 to describe trends in ED visits for substance use. Prior papers have shown: 1) Overall ER volumes decreased during the early months of the pandemic, including visits for emergent conditions; 2) there have been increases in EMS calls for drug overdoses. The authors found that overall ED visits declined during the pandemic, as did visits for substance use disorders (SUD). However, a subset of patients with SUD, those with opioid related visits, showed the lowest decline during the pandemic, and volumes returned to prepandemic levels by the late summer.

The strength of this paper is related to the timely EMR data from a large sample of US ERs. Other than the CDC's syndromic data, it will be at least 1-2 years before national ED data are available to get stats from a more nationally representative dataset. However, there are a number of weaknesses that should be addressed.

- *Thank you for acknowledging the strength of this dataset. In fact, in the time that has elapsed since our original submission, additional emergency medicine groups have submitted electronic data into the national registry and this revision now includes a sample of 258 EDs across 34 states as well as a longer time period from January 1, 2019 through December 31, 2021.*

Larger concerns

1)	Analysis of the primary outcome. The goal of this analysis is to present trends in ED visits for SUDs during the pandemic. The data are presented as counts of SUD visits and subgroups of SUDs. Counts of total ER visits are also provided for comparison on a separate Y-axis in Figures 1-2. The presentation of total ED visit on a different axis can create confusion if this is the only way to visualize SUD visits relative to overall ED visits. The count/volume data are important from a public health and operational standpoint and should be reported, However, it's challenging to make comparisons to what is going on relative to total ED visits within a given month. A suggestion would be to recreate Figures 1 and 2, but make the Y axis counts per 1,000 ED visits. This will give a clearer visualization of what's happening with SUD visits relative to ED volume for a given hospital during that month in the pandemic.

- *Thank you for this observation. In conducting our analysis for this revision we have refined the methods, results and analysis sections to provide count data, an added proportion outcome to address the relative volume issue raised by the reviewer and a regression analysis that provides Incident Rate Ratios (IRRs). We believe that this broader set of outcomes as well as the use of the 3-week moving average ratio ensure that results can be communicated to the reader in a fashion that is more easily interpreted.*

2)	The Discussion and Conclusion do not seem entirely consistent with the results. The data showed (contrary to lay understanding and what is being said in the media) that EDs were not overwhelmed with visits during the pandemic (at least in relation to historical volume) and for the most part visits for SUD went down as well, except for opioid related visits, which returned to baseline. This is stark contrast to the concern that ED visits for substance use and mental health have skyrocketed and that COVID-19 restrictions should be lifted to address this health concern. Reporting trends in SUD visit counts per 1000 EDs would provide a clearer pictured as to whether SUD visits are a bigger relative to clearer relative concern now.

- *In this revision the use of the 3-week moving average ratio better depicts the differences in ED visit patterns for SUD, particularly OUD, in comparison to overall visits and other conditions. We believe that the new figure 1 and 2 address this concern.*
- *We have also revised and extended the discussion to better inform the interpretation of these results.*

Smaller concerns

Introduction

There are some key references that should be included:

-	<https://nam12.safelinks.protection.outlook.com/?url=https%3A%2F%2Fjamanetwork.com%2Fjournals%2Fjamapsychiatry%2Ffullarticle%2F2773768&amp;data=04%7C01%7Carjun.venkatesh%40yale.edu%7Cf3001caac8474c969b4c08d91ef001e3%7Cdd8cbebb21394df8b4114e3e87abeb5c%7C0%7C0%7C637574838440611410%7CUnknown%7CTWFpbGZsb3d8eyJWIjoiMC4wLjAwMDAiLCJQIjoiV2luMzIiLCJBTiI6Ik1haWwiLCJXVCI6Mn0%3D%7C1000&amp;sdata=3OLXweguD%2F4kKwbvXekFCR%2BbDJWCtrpvZJK6Ey6PFLQ%3D&amp;reserved=0>

- *We have added this reference to the introduction as reference #7*

<https://nam12.safelinks.protection.outlook.com/?url=https%3A%2F%2Fjamanetwork.com%2Fjournals%2Fjamanetworkopen%2Ffullarticle%2F2775360&amp;data=04%7C01%7Carjun.venkatesh%40yale.edu%7Cf3001caac8474c969b4c08d91ef001e3%7Cdd8cbebb21394df8b4114e3e87abeb5c%7C0%7C0%7C637574838440611410%7CUnknown%7CTWFpbGZsb3d8eyJWIjoiMC4wLjAwMDAiLCJQIjoiV2luMzIiLCJBTiI6Ik1haWwiLCJXVCI6Mn0%3D%7C1000&amp;sdata=nmqFDr88AfhcbfHrXYXJIfY1tT9v5Ef%2F%2BkCp2DIetmo%3D&amp;reserved=0>

- *We acknowledge the important disparities in SUD outcomes that parallel COVID-19 outcomes. We have added some discussion regarding the important role of the ED in providing access to vulnerable populations and added this reference as well (#26).*

Methods

-	Would be good to include a reference to the representativeness of this cohort of EDs relative to other national data sources of ED visits (e.g % urban vs. rural, %teaching, ownership, ED volume, etc). Details are lacking,

- *We have delineated a section in the methods to describe the dataset with features available including addition of the rural and urban data.*
- *We have also added this sentence for context: “. In general the EDs participating in the ACEP CEDR reflects community, hospital-based EDs of similar regional, case and insurance mix to national datasets with notably fewer academic hospitals”*

Discussion

-	This is really lacking. Need to put findings into context of other studies that have looked at the effect of the pandemic on "deaths of despair" included the references above. For example, could it be possible that out of opioid hospital cardiac arrests are increasing, but many of these patients don't make it to the ER? <https://nam12.safelinks.protection.outlook.com/?url=https%3A%2F%2Fjamanetwork.com%2Fjournals%2Fjamapsychiatry%2Ffullarticle%2F2773768&amp;data=04%7C01%7Carjun.venkatesh%40yale.edu%7Cf3001caac8474c969b4c08d91ef001e3%7Cdd8cbebb21394df8b4114e3e87abeb5c%7C0%7C0%7C637574838440621406%7CUnknown%7CTWFpbGZsb3d8eyJWIjoiMC4wLjAwMDAiLCJQIjoiV2luMzIiLCJBTiI6Ik1haWwiLCJXVCI6Mn0%3D%7C1000&amp;sdata=8b51ZT9YsH5LecQEhxlL4kSu4IzNCL36LunRQhEWU1k%3D&amp;reserved=0>.

- *While the original manuscript was written in a brief format, we have extended the discussion in this revision. We have expanded the explanation paragraph of hypothesis related to these trends.*
- *Specific to this comment, we have added this sentence with the aforementioned references: “In fact, our analysis may underestimate the degree of increased SUD associated harms as EMS data suggests increased out-of-hospital overdose-related cardiac arrests, which may in turn have not resulted in ED visits among people fearful of seeking emergency or hospital care.”*

Could it be possible that the overall rate of SUD visits is stable or declining, but that for Blacks and Hispanics it's actually increasing due to the exacerbation in structural inequities: <https://nam12.safelinks.protection.outlook.com/?url=https%3A%2F%2Fjamanetwork.com%2Fjournals%2Fjamanetworkopen%2Ffullarticle%2F2775360&amp;data=04%7C01%7Carjun.venkatesh%40yale.edu%7Cf3001caac8474c969b4c08d91ef001e3%7Cdd8cbebb21394df8b4114e3e87abeb5c%7C0%7C0%7C637574838440621406%7CUnknown%7CTWFpbGZsb3d8eyJWIjoiMC4wLjAwMDAiLCJQIjoiV2luMzIiLCJBTiI6Ik1haWwiLCJXVCI6Mn0%3D%7C1000&amp;sdata=%2FrckBgOaTLZbIqfxKrWAwfnM0cDPvGEEX0HpxSMMOV8%3D&amp;reserved=0>

- *We have added this reference to the paper and aknowledged the importance of disparities in this revision along with the above reference: “with opioid overdoses exhibiting racial and ethnic disparities as well,“…“while we acknowledge the likely important interaction between race and ethnicity and these observed trends, the ACEP CEDR registry does not currently capture this data reliably and future work should explore whether observed changes in visitation trends reflect widening disparities in access to SUD care for disadvantaged populations.”*
- *Unfortunately, race and ethnicity data is not currently reliably captured in the ACEP CEDR registry, and we have aknowledged this limitation as an area for future inquiry.*

-	Or could the pandemic have forced some policy changes that make it easier to take care of patients with SUD outside the hospital (eg ability to initiate buprenorphine over the phone)?

- *We have aknowledged this as well in the limitations since we actually observe increased much higher ED visit rates for OUD in comparison to other SUD despite OUD being the most amenable to such telemedicine interventions.*

General comment - I appreciate brevity, but it seems like this paper is stuck somewhere between a research letter and full-length manuscript. Would suggest going in one direction or the other, probably towards adding more details.

- *As aforementioned we had originally tried to frame the work as a research letter/brief report; however in this submission have substantially lengthened the paper into a full-length manuscript.*

Reviewer #2: Thank you for allowing me to review this manuscript. Overall, this is an extremely important topic and terrific source of data, although I have major concerns about the study objectives and design. The presentation of the methods is unstructured and rushed, leading to confusion on the findings presented. The manuscript could be greatly improved with a more clear hypothesis, better description of the study outcomes, better explanation of the chosen analysis, and clarity around the 'sensitivity analysis.' I also do not believe that the chosen analysis is appropriate for the questions being asked, but it is hard to tell because of the lack of detail on what is being presented. Most importantly, the study could benefit from a hypothesis on what the authors expect to occur for ED SUD visits during COVID.

- *In this revision we have added structured subheadings to the methods section as well as addition of limitations to provide more clarity and readability*
- *As aforementioned, we have also clarified that while the primary outcome is ED visits counts, we have analyzed counts in 3 ways to provide a comprehensive picture of trends including total counts, proportional changes, 3 week-moving average ratio, and incident rate ratios.*
- *This work is intended to be descriptive and as such we did not have any hypothesis except perhaps that ED visitation for SUD would change over time similar to all visits. We do not seek to statistically test that hypothesis as that is not a central question for clinicians or policymakers. Instead we believe that the refined outcomes definitions and results of this revision will address this concern.*

Abstract

-	Style edits throughout for confusing language, such as "understanding trends in ED utilization within the ED"

- *We have made substantial edits to the abstract to improve readability including revision of that phrase.*

-	Do the authors have a clear hypothesis for whether ED visit rates for SUD will increase or decrease due to COVID? This may help clarify framing but also purpose.

- *As aforementioned, this observational study was intended to be descriptive and not hypothesis based. We have added an explicit objective statement to the abstract and manuscript to address this need for clarity.*

-	The Methods section in the Abstract needs more structure and clarity. It is not clear what the primary outcome or primary analysis is, or how geography or COVID rates fit into the analysis. Is the sensitivity analysis actually a main analysis, given that this accounts for variation in geography and COVID rates?

- *We have added subheadings to the Methods section to provide structure including designation of primary and secondary outcomes as well as the primary and secondary analysis.*
- *The geographic analysis is a secondary analysis and presented as such in this revision.*

-	The Findings also read as a bit of a surprise, due to lack of structure. For example, there is no mention of stratifying analysis by "early" or "late" pandemic, but in the same sentence only one IRR is reported - presumably for the entire study period?

- *In this revision we have removed the term “late” and tried to report results based on the dates visible in the figures and tables. We have also provided date definitions to the term “early” when used.*

-	The conclusion is not supported by the results - if rates are decreased, why are more resources needed? I do not dispute the conclusion, but the reason is not supported by the analysis described.

- *We believe our findings, particularly now using 3 week moving average ratios depict an increase in ED visit rates for SUD relative to other conditions and an absolute increase for OUD that warrants allocation of more resources.*
- *We have further refined the conclusions to build off the newly expanded discussion section.*

Introduction

-	The end of paragraph 1 is confusing, and makes it seem as if the paper will discuss COVID ED visits for patients with SUD (rather than SUD ED visits for patients during the COVID era)

- *We intended to allude to the interaction between COVID and SUD but recognize this confusion—we have removed this from the introduction and re-introduced the reference and topic within the discussion.*

-	The important problems and tensions discussed in this paper are crammed into a few sentences in the second paragraph - I would consider delving a little further into why this is important, and particularly what the hypotheses are.

- *We have addressed this be expanding upon the role of the ED and these problems in the discussion instead of in the introduction.*
- *Also, we have added clear objective statements for this descriptive work.*

-	It makes the framing much clearer (and the results more interpretable) if you anticipate a relative rise in ED visits for SUD (decreased access to outpatient treatment leads to more ED use) or decrease in ED visits for SUD (people with SUD still afraid to come to the ED / overdosing alone, etc) - either of which indicates a problem, just a different one.

- *Rather than make speculative hypothesis, we believe the importance of this analysis is observational and descriptive. To that end we have added clear objective statements and the newly refined outcome definitions will likely address some of the confusion in interpreting trends.*

-	It is unclear from the introduction whether the authors are going to analyze changes in SUD visit rates compared to all ED visits (relative change) or an absolute change in ED SUD visits.

- *The primary study outcome is absolute counts, however these counts can be analyzed in both absolute and relative manners, which we have now specified in the methods, results and supplement.*

Methods

-	This section includes results, which is a bit confusing.

- *We have had added subheadings for clarity*
- *In addition, the only “results” that appear in the methods are the description of the registry (not necessarily the dataset) which we believe is better placed in the methods under the “dataset” subheading*

-	What is the main analysis? Is it the trends using LOWESS, and are you comparing slopes or trends between groups? What is going into the Poisson regression models? Why would the geographic and temporal factors due to COVID be in the sensitivity analysis rather than the main analysis? The methods are very rushed and not particularly clear - this essentially is no more detailed (or helpful) than the abstract.

- *We have added explicit objective statements*
- *We have also created an analysis section indicating the primary analysis is inclusive of descriptions of each outcome. The primary analysis is inclusive of regression models utilized*
- *The geographic analysis is a secondary analysis and has been described as such in the analysis section, given a distinct paragraph in the results as well as the discussion.*

Results

-	The results section makes several claims which do not have support. Are the authors making qualitative claims about trends? For example, "In comparison, ED utilization for non-alcohol and non-opioid substances have declined more precipitously in the early pandemic period and have remained lower as well in subsequent months." I don't see a statistic that justifies the 'more precipitous' decline - like a time series analysis or even a pre-post comparison of slopes.

- *In this revision we use more specific language in the results section to describe updated including additional graphs in the manuscript and supplement.*

-	The key finding of the article - is the fact that declines in SUD and mental health visits are lesser than overall declines - does not have any statistical or other basis to support that claim - or if it does than I do not see or understand the methods that have led the authors to that conclusion. In the table, the AUD/SUD/OUD visits are included in different models than MI / stroke - so it is not possible to compare across conditions.

- *In this revision we make this comparison easier to draw by 1) use of the 3 week moving average ratio to depict the comparison graphically and 2) the use of segmented regression models that allow for the estimation of IRRs which can be interpreted in comparison to each other.*

Reviewer #3: I confine my remarks to statistical aspects of this paper. These were very well done and I recommend publication.

Peter Flom

- *Thank you – we believe we have addressed these comments as above.*
